# A comic-based body image intervention for adolescents in semi-rural Indian schools: A randomised controlled trial

**DOI:** 10.1016/j.ijchp.2025.100546

**Published:** 2025-01-26

**Authors:** Helena Lewis-Smith, Latika Ahuja, Farheen Hasan, Caterina Gentili, Paul White, Phillippa C. Diedrichs

**Affiliations:** a Centre for Appearance Research, University of the West of England, Frenchay Campus, Coldharbour Lane, Bristol, BS161QY, UK; b Applied Statistics Group, University of the West of England, Frenchay Campus, Coldharbour Lane, Bristol, BS161QY, UK

**Keywords:** Body image, India, Adolescents, Intervention, School, Randomised Controlled Trial

## Abstract

Adolescents in India experience body dissatisfaction and its associated adverse impacts on physical and mental health and gender equality. However, evidence-based interventions are scarce. Mental health interventions worldwide have traditionally relied upon delivery by expert providers. However, this prevents scalability, particularly in rural settings, where resources are often lacking. Therefore, this study evaluated the efficacy of a low-resource teacher-delivered mixed-gender comic-based body image intervention among adolescents in semi-rural Indian schools. A randomised controlled trial was conducted among 2631 students (50 % girls; classes 6 to 8; Mage = 12.03 years, *SD* = 1.22) across 41 schools around the Jaipur district in Rajasthan. Each school was randomly allocated to receive six comic-based intervention sessions (*n* = 1347) or lessons-as-usual (*n* = 1284; control). The primary outcome of body esteem and related secondary and exploratory outcomes assessing mental health and gender stereotyping were assessed at baseline, 1 week-post-intervention, and 3-months follow-up (ClinicalTrials.gov, NCT04317755). Linear Mixed Model analyses revealed that compared to the control group, intervention students reported significantly higher body esteem and skin shade satisfaction, and significantly lower eating pathology, internalisation of appearance ideals, and gender stereotyping, with all effects maintained at follow-up. Compared to control group, boys in the intervention group also demonstrated significantly higher body image-related life engagement and body hair satisfaction at follow-up. Both students and teachers indicated high intervention acceptability via quantitative and qualitative findings. These findings present the first effective teacher-delivered school-based body image intervention in India, which can be implemented at scale using minimal resources, and thus indicates promise regarding broader dissemination across urban and rural settings.

## Introduction

Body dissatisfaction is a public health issue among adolescents globally, including in India, the world's largest lower-medium-income country (Al Sabbah et al., 2009). Whilst prospective studies are currently absent, research suggests that body image concerns increase from childhood to adolescence in India, at which point approximately 66–78 % of this group report dissatisfaction with their appearance (Ganesan, Ravishankar, & Ramalingam, 2018; Mondal, Ghosh, Maji, & Ray, 2021; Sánchez-Castillo et al., 2019; Singh, Parsekar, & TV, 2016). This warrants concern, due to associated adverse consequences, including depression, disordered eating, and the use of harmful skin lightening products (Ganesan et al., 2018; Jayan, 2021; Shroff, Diedrichs, & Craddock, 2018; Singh et al., 2016). Eating disorder symptomology tends to develop around late adolescence, with a third of individuals at risk for a full threshold eating disorder (Singh et al., 2016; Vaidyanathan, Kuppili, & Menon, 2019). This is also the peak time for depression, with Indian youth suicides the highest globally (Capaldi & Stoolmiller, 1999; Patel, Flisher, Hetrick, & McGorry, 2007). Further, skin shade dissatisfaction, a salient appearance concern, leads to the use of dangerous skin lightening products (Peltzer, Pengpid, & James, 2016). This maintains long-established social inequalities due to the cultural “currency” of skin shade within India, whereby lighter skin tone has been traditionally associated with higher social position, privilege, and power (Negi, Abdul Azeez, & Rani, 2024; Shroff et al., 2018). This insinuation is a consequence of ‘colourism’, a form of prejudice that discriminates against people with darker skin tones and can occur between members of the same ethnic group (Hunter, 2002)^1^. Experiencing colourism is associated with greater skin shade dissatisfaction, which in turn has been related to the increased use of harmful skin lightening products (Craddock et al., 2023; Harper & Choma, 2019). Collectively, this highlights body dissatisfaction as a public mental health and social justice issue among Indian adolescents, stressing the urgency for interventions for this group.

Schools constitute a promising avenue for a cost-effective delivery of universal adolescent body image interventions at scale (Chua, Tam, & Shorey, 2020; World Health Organisation, 2021; Yager, Diedrichs, Ricciardelli, & Halliwell, 2013b). This is particularly the case in India, where less than 1 % of the healthcare budget is allocated to mental health (Math et al., 2019), with only 10 % of adolescents having access to these services (Patel et al., 2007). Further, the Indian government, teachers, parents, and students have called for the provision of school-delivered mental health support (Parikh et al., 2019). Additionally, schools provide the opportunity to simultaneously target both girls and boys, who experience an overlap in risk factors for body image concerns (e.g., Paterna, Alcaraz-Ibáñez, & Sicilia, 2021) and thus might benefit from the same aetiologically based interventions. Moreover, research indicates that school-based body image interventions can be equally as effective when delivered in single- or mixed- gender class settings, with both genders actually expressing a preference for mixed-gender settings (Dunstan, Paxton, & McLean, 2017, Yager, Diedrichs, & Drummond, 2013a). Considering all of this, we previously evaluated ‘Confident Me’, the first evidence-based mixed-gender body image intervention in Indian schools (Garbett et al., 2023, Lewis-Smith et al., 2023). This five-session psychologist-delivered intervention was delivered to students aged 11–14 years across private schools in urban Delhi, with its effects compared with a lessons-as-usual control. Findings revealed relative improvements among students who received the intervention compared with those in the control arm in relation to body image, disordered eating, and negative affect (Garbett et al., 2023, Lewis-Smith et al., 2023). Whilst these findings indicate a promising school-based body image intervention, it may not be appropriate for implementation in more rural and lower socio-economic areas of India, where body dissatisfaction and problematic gender stereotypes, are salient (Batra & Reio Jr, 2016; De Sousa, Mohandas, & Javed, 2020; Johnson, Balasubramanya, & Britto, 2015; Mondal et al., 2021). For example, ’Confident Me’ is centred around PowerPoint and video stimuli, but rural schools often lack digital tools (Naik, Chitre, Bhalla, & Rajan, 2020; Tyagi, 2018). Further, delivery by a psychologist is costly and may not be affordable for rural schools, and unsurprisingly, there have been calls to task-shift mental health interventions to help facilitate scalability in low-middle income countries (Kazdin, 2019). This highlights the need to develop a low-resource body image intervention that can be disseminated at scale across schools in more rural regions of India.

Storytelling interventions are efficacious in improving body image in high-income countries (Dohnt & Tiggemann, 2008; Mills & Osborn, 2013), and the modality is well-placed to target hard-to-reach groups, including rural adolescents (Brouzos, Vassilopoulos, & Moschou, 2016). Comic books (or comics) are a particularly fitting medium, given their popularity in depicting the stories of Indian epics (e.g., ‘Ramayana’; McLain, 2009). Further, their heavy use of imagery (visual cues to the text) makes them well-suited to rural regions, where literacy rates are lower (Abdulrahaman et al., 2020; Cohn, 2014). Thus, a Hindi comics-based body image intervention was co-created with UNICEF, the Dove Self-Esteem Project (DSEP; a social purpose industry initiative for Unilever brand Dove), the first three and last authors, and BBC Media Action, for adolescents in rural India (Lewis-Smith et al., 2022). Based on the effectiveness of ‘Confident Me’ for both girls and boys in the Indian school setting (Garbett et al., 2021, Lewis-Smith et al., 2023), the comics-based intervention was designed to be delivered in mixed-gender classes. This was also deemed important due to both genders constituting an important part of each other's social environment that can influence appearance pressures and gender stereotyping, and thus provides the opportunity for them to learn from one other (BBC Media Action, 2019; Yager et al., 2013a).

Prior to the present research, an acceptability study was conducted in government schools in Rajasthan, and findings revealed positive perceptions relating to the intervention (Ahuja et al., 2022). Therefore, the present study aimed to evaluate the efficacy of the intervention in a large-scale randomised controlled trial. Whilst the focus was on providing body image support to adolescents in more rural regions of India, the present study was conducted in semi-rural India. Semi-rural regions occupy approximately 27 % of the country, and tend to have a lower population density and less developed infrastructure than urban areas, but have a higher population density and more developed infrastructure than rural areas. Consequently, compared with adolescents in rural India, those in semi-rural India are more likely to have access to a smartphone and therefore greater exposure to appearance ideals and related messaging via digital media, which is associated with body image concerns (Sindakis & Showkat, 2024; Singh et al., 2016). Further, whilst there tends to be a greater number of schools in semi-rural Indian than rural India, the resources are more limited than in urban schools. Consequently, a pragmatic decision was adopted to test the intervention in a semi-rural region of India. Nonetheless, if found to be effective in improving body image, the intervention will be disseminated by UNICEF with support from Dove across schools in eight states of India, thus having the potential to improve body image among millions of young people.

## Materials and methods

### Trial design and setting

A pragmatic, single-blinded, parallel two-arm randomised controlled trial (RCT) was conducted across 42 government schools in semi-rural Rajasthan, India. Participants completed paper-based assessments at baseline, post-intervention, and at 3-month follow-up. The trial was approved by the authors’ university ethical committee (HAS.18.01.074), registered at ClinicalTrials.gov (NCT04317755), and the protocol published (Lewis-Smith et al., 2022). The trial was conducted in line with CONSORT 2010 guidelines.

### Recruitment and participants

Permission was sought from Rajasthan's Department of Education to invite government schools in semi-rural regions to participate. Eligibility included being co-educational, teaching in Hindi, and including years 6–8 (aged 11–14 years). This age group was selected due to experiencing a high prevalence of body dissatisfaction (Mondal et al., 2021).

#### Sample size

The sample size was calculated based on the earlier evaluation of an Indian school-based body image intervention, which indicated a range of effect sizes for outcome measures with standardised effects in the small to medium range and exceeding an effect size of *d* = 0.2, which would minimally be of interest to public health policy makers (Lewis-Smith et al., 2023). For a lower bound of *d* = 0.2, a sample size of *n* = 540 boys and *n* = 540 girls per arm would have 95 % power to detect an effect separately for each gender (two-sided, alpha = 0.05). To account for 10 % loss, the sample size was inflated by (540/10 =) 54 of each gender per arm, and to account for 10 % loss within this additional recruitment, a further inflation of (54/10 =) 6 to give a target sample size of 600 boys and 600 girls per arm, totalling 1200 per arm (intervention versus control). Therefore, we aimed to recruit a sample of 2400 students divided across school year 6,7, and 8, and gender.

#### Randomisation

**Fig. 1:**
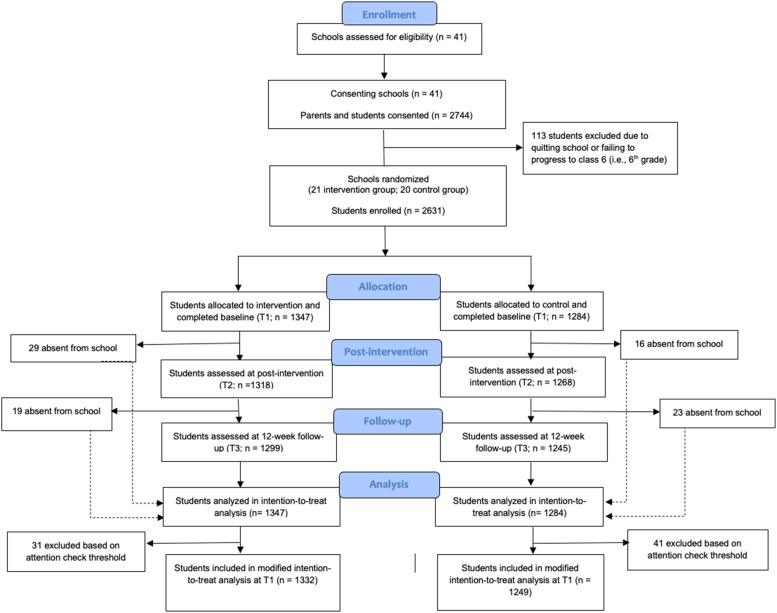
CONSORT 2010 flow diagram for the study.

Eligible schools were randomised to either the intervention or control arm by an external blinded researcher via computer-generated randomisation (see Fig. 1). Randomization was conducted at the school level to avoid spillover effects due to communication about the intervention to the control group. Schools were randomised in a 1:1 ratio using minimization with a residual error. The minimization factors were (i) number of students at grade 6, 7 or 8 and (ii) proportion of boys at grade 6, 7, and 8. The first 8 schools were allocated using blocked randomization and thereafter probabilistic minimization occurred with the probability of minimizing set to 0.8. Whilst school headteachers were aware of whether their school had been randomised to the intervention or control condition, students were not aware of the research design or that there was an “alternative”. Students in both conditions were told that the study aimed to help young people feel confident in themselves and that they would be asked to complete three surveys. However, those in the intervention condition were also told that they would be reading comics in their classes.

### Intervention

The comics were developed using a multi-stage rigorous approach, whereby the authors, UNICEF, BBC Media Action, and DSEP brought their unique expertise into a collaborative and iterative process. The first stage involved all parties attending an in-person two-day workshop to share their knowledge with one another. Given that systematic reviews indicate the most promising body image interventions to be theory-based (e.g., Ciao, Loth, & Neumark-Sztainer, 2014; Schwartz et al., 2019; Yager at al., 2013b), an aetiological approach was employed, whereby established influences on body dissatisfaction were targeted. This involved the group coming up with themes for the comics, with each of the six comics targeting an influence, including gender stereotyping, pressure to conform to appearance ideals, media pressure to alter appearance, body talk, and appearance-based comparisons.

The second stage involved BBC Media Action proposing brief storylines for each comic, which were subsequently discussed in online meetings among all four parties in relation to their incorporation of the specific risk factor, creative execution, and relevance to a rural setting. The comics were consequently revised in an iterative cycle based on this feedback, and this was followed by the development of two interactive activities for each respective comic. This next stage involved executing an acceptability study among 35 students and nine teachers from Hindi medium schools, who were asked to read the comics and associated activities independently (Ahuja et al., 2022). Interviews were conducted with these teachers and students to explore their feedback and suggestions for improvement, and they also completed a brief questionnaire to quantitatively capture their perceptions of the intervention. Findings revealed positive feedback and perceptions relating to the intervention, with valuable insights on intervention optimisation (e.g., making concepts less nuanced and more explicit, changing the format of the activities), which were implemented in the final version of the comics. For more details relating to the development of the comics and the acceptability study, see Ahuja et al. (2022) and Lewis-Smith et al. (2022).

Each comic included a story centred around three teenagers who solve a different case related to body image in a small imaginary town (e.g., a boy stealing his friend's elephant because he was jealous of his appearance; a girl not taking part in a sports day due to the risk of getting darker in the sun), and consequently teach the residents a lesson pertaining to body image (e.g., how they should not compare their looks to others, how it is impossible to attain appearance ideals). Each story is followed by two interactive activities (e.g., identifying whether certain activities are usually done by a man, a woman, or both, in their home; completing statements related to costs associated with comparing oneself to others; giving peers a non-appearance-based compliment) to help students internalise the story's messages (see Ahuja, Hasan, Diedrichs, & Lewis-Smith, 2022, Lewis-Smith, Hasan, Ahuja, White, & Diedrichs, 2022 for details relating to intervention content). Each session commenced with the teacher reading the comic with the class. When finished, the teacher asked the students for their feedback on the comic and clarified any words and sentences they struggled to understand. This first part lasted approximately 30 min. Next, the teacher facilitated the two activities. However, they prioritised the first activity and facilitated the second activity only if there was time^2^. This second part lasted approximately 15 min. Most classes did not have time to complete the second activity. Once these were discussed as a class, the session ended. Sessions occurred during class time (1 comic per 45 min lesson; 6 lessons in total), with teachers delivering the intervention using an intervention guide, as this has been deemed helpful by Indian teachers previously (Garbett et al., 2021). All materials were developed in Hindi. See Ahuja et al. (2022) and Lewis-Smith et al. (2022) for further intervention details.

Teachers received a two-day training course from an Indian research agency, who had received a two-day training course from the authors. This ‘train-the-trainer’ approach has proved effective and scalable in previous school-based body image research (Greif, Becker, & Hildebrandt, 2015). The teacher training covered the theoretical basis of the intervention (i.e., Tripartite Influence Model of Body Image; Keery et al., 2004), including risk factors for body dissatisfaction and associated consequences. Subsequently, the teachers were familiarised with the comics and associated activities, and participated in role plays to practise delivering the intervention. Finally, they were given hints and tips for delivering the sessions (e.g., being a body confident role model, making the sessions as interactive as possible) and practise answering challenging questions that could be posed by the students (e.g., “This is a girl's issue. Why do we boys have to do this?”, “Surely being skinny is healthy though?”).

### Control condition

An assessment-only control condition was employed to reduce threats to internal validity (Mohr et al., 2009). Students in this condition received their usual lessons and completed the assessments at the same time points as students in the intervention condition.^3^

### Procedure

Written informed consent was first sought from headteachers for their respective school's participation. In the context of anonymous public health research, the Indian Council of Medical Research's guidelines state that parental consent is sought at the school's discretion. However, parents were given two weeks to provide opt-out consent for their child to participate in the study. On the day of baseline data collection, a researcher read the participant information sheet and consent form aloud to students in the classroom to ensure comprehension. Once students were given the opportunity to ask questions, they were asked to indicate written assent. Once all consent procedures were completed, students were given the baseline (T1) paper-based self-report paper survey. Due to concerns regarding students’ literacy skills and the potential for students to end up copying each other, we employed a data collection approach that was considered helpful in our previous research conducted in Indian school settings (e.g., Garbett et al., 2021, Lewis-Smith et al., 2023). This involved having the researcher read through each question aloud and asking whether students understood what was being asked. This provided the opportunity to explain anything that students struggled to understand, with students subsequently completing the respective question on their own before the whole class moved onto the next question. The data collection session took approximately 40 min. Further outcome assessments were administered in a similar manner at post-intervention (T2) and 3-month follow-up (T3).

### Measures

#### Efficacy measures

The primary outcome was body esteem. Secondary outcomes included internalisation of appearance ideals, a common risk factor for body image concerns among adolescents in high-income countries, which has been identified as a correlate of body dissatisfaction in India (e.g., Kakar et al., 2023). Other secondary outcomes encompassed eating pathology, body image related life engagement, and positive and negative affect, which are recognised psychosocial consequences of body image among young people in high-income countries, and have received cross-sectional support in India (e.g., Dove, 2017; Ganesan et al., 2018; Kakar et al., 2023). All measures assessing primary and secondary outcomes were validated English-language measures that have been widely used among adolescents and underwent subsequent rigorous validation in Hindi by the present authors using recommended steps (Swami, 2019)^4^. These included the following original scales: the Body Esteem Scale for Adults and Adolescents (Mendelson et al., 2001), the Child Eating-Disorder Examination Questionnaire (Kliem et al., 2017), the Internalisation-General subscale of the Sociocultural Attitudes Towards Appearance Questionnaire-3 (Thompson et al., 2004), the Body Image Life Disengagement Questionnaire (Atkinson & Diedrichs, 2021), and the Positive and Negative Affect Schedule for Children (Ebesutani et al., 2012).

We were also interested in investigating additional exploratory outcomes that have been indicated as salient issues and related to body image among Indian young people (e.g., Adithyan et al., 2018, Patel et al., 2021; Peltzer et al., 2016; Phadke, 2017) but currently lack validated measures for their assessment. These included a single-item measure to assess appearance-based teasing that was constructed specifically for a previous school-based trial (Diedrichs et al., 2021), a purposely constructed 10-item scale to assess the endorsement of gender stereotypes, a purposely constructed single-item scale to assess skin shade satisfaction, and a purposely constructed single-item scale to assess body hair satisfaction. Whilst these measures did not undergo validation, they were assessed for comprehension via cognitive interviews with 12 Hindi-speaking Indian adolescents (50 % girls) aged 11–14. Since publication of the original protocol (Lewis-Smith et al., 2022), the decision was made to add two image-based outcome measures of skin shade satisfaction and body size satisfaction to complement other text-based measures (see Table 1 for details). This was informed by the cognitive interviews and further discussions with stakeholders regarding concerns about students’ low literacy levels.

**Table 1:** Outcome measures and internal consistencies (Cronbach's alpha for the present sample).

Outcome	Scale Description	Girls: α (*n* = 1316)	Boys: α (*n* = 1315)
*Primary Outcome*			
Body esteem	Body Esteem Scale for Adolescents & Adults (Mendelson et al., 2001), validated among Indian adolescents (manuscript in preparation). Seventeen items, mean score range 1–5. Higher scores indicate greater body esteem, which is desirable. Example item: *‘I think I have a good-looking body’ (1 = never – 5 = always).*	0.76	0.77
*Secondary Outcomes*			
Eating pathology	Child Eating-Disorder Examination Questionnaire (Kliem et al., 2017), validated among Indian adolescents (manuscript in preparation). Eight items, Example item: *‘How unhappy have you been with your weight?’ (1 = not at all – 5 = very much so).*	0.76	0.79
Internalisation of appearance Ideals	Internalisation-General subscale of the Sociocultural Attitudes Towards Appearance Questionnaire-3 (Thompson et al., 2004) validated among Indian adolescents (manuscript in preparation). Nine items, mean score range 1–5. Higher scores indicate greater internalisation of appearance ideals, which is not desirable. Example item: *‘I would like my body to look like the people in movies’ (1 = totally disagree – 5 = totally agree).*	0.86	0.85
Body image related life engagement	Body Image Life Disengagement Questionnaire (Atkinson & Diedrichs, 2021), validated among Indian adolescents (manuscript under review). Nine items, mean score range 1–4. Example item: *‘In the past two weeks, have you stopped yourself from going to the doctor because you felt worried about the way you looked?’ (1 = hasn't stopped me at all – 4 = stopped me all the time).* Higher scores usually indicate greater life disengagement. For the present, all items were reverse scored, whereby higher scores indicate less life disengagement due to body image concerns, which is desirable.	0.77	0.78
Positive affect	Positive Affect subscale of the Positive and Negative Affect Schedule for Children (Ebesutani et al., 2012), validated among Indian adolescents (manuscript in preparation). Four items, mean score range 1–5. Higher scores indicate greater positive affect, which is desirable. Example item: ‘*How often have you felt happy over the past two weeks?’ (1 = not at all – 5 = very much*).	0.74	0.74
Negative affect	Negative Affect subscale of the Positive and Negative Affect Schedule for Children (Ebesutani et al., 2012), validated among Indian adolescents (manuscript in preparation). Five items, mean score range 1–5. Higher scores indicate greater negative affect, which is not desirable. Example item: ‘*How often have you felt sad over the past two weeks?’ (1 = not at all – 5 = very much*).	0.62	0.64
*Exploratory Outcomes*			
Endorsement of gender stereotypes	Purposely constructed measure, ten items, mean score range 1–5. Higher scores indicate greater endorsement of stereotypes related to how girls and boys should behave, which is not desirable. Example item: *‘Girls should not go outside to play when it's dark’ (1 = strongly disagree – 5 = strongly agree).*	0.75	0.75
Skin shade satisfaction	i) Purposely constructed single-item measure: ‘*How satisfied or dissatisfied are you with the colour of your skin?’ (1 = very dissatisfied – 5 = very satisfied)*. Higher score indicates greater skin shade satisfaction, which is desirable. ii) Purposely constructed skin shade rating scale. Nine images reflecting different skin shades ranging from darker (1) to lighter (9)^1^, from which respondents select both their perceived skin shade and their desired skin shade. The absolute discrepancy score between the two numbered images indicates the degree of skin shade dissatisfaction (range 0–8). Lower scores indicate greater skin shade satisfaction, which is desirable.	N/A	N/A
Body hair satisfaction	Purposely constructed single-item measure: ‘*How satisfied or dissatisfied are you with the amount of hair on your body?’ (1 = very dissatisfied – 5 = very satisfied)*. Higher score indicates greater body hair satisfaction, which is desirable.	N/A	N/A
Appearance-based teasing	Single-item measure (Diedrichs et al., 2021): ‘*How often have you been teased about the way you look?’(1 = never – 5 = always)*. Higher score indicates higher frequency of experiencing appearance-based teasing, which is not desirable.	N/A	N/A
Body size satisfaction	Child Figure Rating Scale (Tiggemann & Wilson‐Barrett, 1998). Nine images of adolescents ranging from small (1) to large (9), from which respondents select both their perceived figure and their desired figure. The absolute discrepancy score between the two numbered images indicates the degree of body size dissatisfaction (range 0–8). Lower scores indicate greater shape/size satisfaction, which is desirable.	N/A	N/A

1 The range of skin shades were taken from a skin shade chart regularly used in Indian market research, including by Unilever.

All outcome measures and associated internal consistencies for the present sample are displayed in Table 1, and further detailed in Lewis-Smith et al. (2022)^5^.

#### Fidelity measures

As per previous research (Garbett et al., 2021, Lewis-Smith et al., 2023), intervention fidelity was assessed across schools and sessions. Of the total 254 sessions, 127 (50 %) were audio-recorded, of which 85 (33.3 %) were assessed on perceived teacher competency (percentage reflecting how well teachers delivered the sessions) and adherence to the teacher guide (percentage reflecting the extent to which teachers completed outlined key actions). A selection of the 22 observed sessions (25 %; Diedrichs et al., 2021) were used to assess inter-rater reliability via intra-class coefficients (ICC).

#### Acceptability measures

As per similar trials (Diedrichs et al., 2021, Garbett et al., 2023), intervention acceptability was assessed both quantitatively (Table S1) and qualitatively (Tables S2 and S3) at post-intervention. The quantitative assessment asked students (*n* = 1318) to respond to six positively worded statements (e.g., “I enjoyed the sessions”, “I understood what was being taught in the sessions”) using a 5-point Likert scale (1 = Strongly Disagree; 5 = Strongly Agree). Responses were subsequently collapsed into three response categories during the analysis (Agree, Neutral, and Disagree). The quantitative assessment among teachers (*n* = 50) asked them to respond to 10 positively worded questions (e.g., “Did you enjoy delivering the sessions?”, “Do you think the sessions helped adolescents with their body confidence?”) using a 5-point Likert scale (1 = Not at all; 5 = Very much). Responses were subsequently collapsed into three response categories during the analysis (Yes, Neutral, and No).

With regard to qualitative assessments, six focus groups were conducted with four students (24 students in total) in a classroom. In each focus group, the researcher asked the students questions relating to their interest in the comics (e.g., “Did you find the comics fun/interesting to read? Why/why not?”), the relevance of appearance concerns (e.g., “Did you find the appearance concerns in the stories relevant?”), learnings (e.g., “Were there any key learnings you found particularly relevant and helpful?”), and perceived benefits (e.g., “Has reading these comics changed the way you feel about yourself or your appearance?”). Students were also asked about challenges (e.g., “Were there any (other) things you found difficult to understand in the comics or activities?) and future recommendations relating to the comics (e.g., “Can you think about how the sessions might be improved for future students?”). With regard to teachers, two focus groups were conducted with six teachers (12 teachers in total) in a classroom. The researcher asked the teachers questions relating to training and preparation (e.g., “How did you find the teacher training session?”), perceived benefits of the comics (e.g., “How do you think students found the comics and the programme?), student engagement (e.g., “How did students engage with the comics and activities?”), delivery of the programme (e.g., “How did you find delivering the sessions?”), and their views of the Teacher Guide (e.g., “ Was the Teacher Guide easy to follow while delivering the sessions?”). Finally, they were asked about challenges (e.g., “Were there any (other) things that you or students found difficult throughout the sessions?") and future recommendations relating to the comics (e.g., “What should be taken into account for future delivery of these sessions?").

#### Validity measures

To examine the validity of the efficacy findings, intervention students were also asked to recall any key learnings from the sessions in the post-intervention questionnaire. These were analysed using descriptive statistics, whereby the frequency of responses that reflected key learning outcomes for each respective session was calculated (Table S3).

### Statistical analysis

Analyses were executed using SPSS 28 and R studio. The data analyst was blinded to condition allocation to avoid interpretation bias. Concealment of condition allocation was lifted when running exploratory analyses. Participants had to answer 75 % of seven attention check items correctly to be included in the analyses.

#### Hypotheses testing and post-hoc analyses

The effect of the intervention on primary and secondary continuous outcome measures was examined by running Linear Mixed Models (as described in Lewis-Smith et al., 2022). The models included baseline measures at T1 as a covariate and three main effects: randomised arm as a two-level between-subjects factor, study phase (T2, T3) as a two-level repeated measures factor, and gender as a two-level between subjects factor. The statistical model also included six two-way interactions (randomised arm*study phase, randomised arm*gender, study phase*gender, covariate*randomised arm, covariate*study phase, covariate*gender) and one three-way interaction between randomised arm, gender, and study phase. For effect size estimation, partial eta squared was calculated (whereby 0.01 was considered a ‘small’ effect size`, 0.06 a ‘medium’ effect size`, and 0.14 a ‘large’ effect size`; Cohen, 1988).

For each outcome, pre-planned ANCOVAs were conducted to verify the effect of randomised group at each of T2 and T3 separately. All post-hoc ANCOVAs were run first ignoring gender, and then separately for girls and boys. Ordinal exploratory outcomes were compared between randomised arms separately at T2 and T3 using ordinal logistic regressions, including baseline measurement as a covariate and randomised group as the independent variable.

### Acceptability analysis

All acceptability analyses were conducted separately for teachers and students. Quantitative data analysis was analysed using descriptive statistics, including frequencies and means. Focus group discussions were analysed using qualitative codebook thematic analysis (Fereday & Muir-Cochrane, 2006), which involves creating codes and themes *a priori* (MacQueen et al., 1998). Initially, two coders generated a codebook from the raw data, and then went on to identify analytically relevant themes and sub-themes. Whilst the student and teacher data were coded independently, the similarity of the codes led to the data being combined and analysed simultaneously. Engaging in reflexive analysis, both coders discussed and agreed on the final themes.

## Results

### Recruitment and sample characteristics

A total of 2631 students (intervention *n* = 1347; control *n* = 1284) were recruited across 41 schools between August and December 2021, of which 2581 participants were included in the final analyses (see Fig. 1). There was an equal distribution of gender (50.02 % girls) and age (*M* = 12.03 years, *SD* = 1.22) across the sample. Nearly all were Hindu (95.52 %), with most speaking an additional language other than Hindi at home (66.66 %), and having parents educated up to high school level (father, 67.96 %; mother, 50.28 %). Whilst nearly all students had access to a mobile phone (91.03 %), less had a computer (14.33 %) or television (64.19 %) at home (Table 2). Regarding baseline comparisons, the intervention group were significantly more likely to speak another language in addition to Hindi, whilst the control group were more likely to have a mobile phone (*p* < 0.05).

**Table 2:** Sociodemographic characteristics of participants (at baseline).

	Control (*n* = 1284)	Intervention (*n* = 1347)	Total (*n* = 2631)	t/x^2^ value	*p value*
**Age***M* (*SD*)	12.03 (1.22)	12.07 (1.21)	12.03 (1.22)	–1.533	0.13
**Gender***n (%)*					
Girls	657 (51.17 %)	659 (48.92 %)	1316 (50.02 %)	1.330	0.26
Boys	627 (48.83 %)	688 (51.07 %)	1315 (49.98 %)		
**Class (i.e., Grade)***n (%)*					
6th class	401 (31.23 %)	380 (28.21 %)	781 (29.7 %)	2.80	0.25
7th class	407 (31.7 %)	430 (31.92 %)	837 (31.81 %)		
8th class	473 (36.84 %)	525 (39.0 %)	998 (37.93 %)		
**Religion***n (%)*					
Hindu	1243 (96.81 %)	1270 (94.3 %)	2513 (95.52)	9.41	0.52
Muslim	16 (1.25 %)	34 (2.52 %)	50 (1.9 %)		
Sikh	5 (0.4 %)	2 (0.1 %)	7 (0.3 %)		
Christian	-	2 (0.15 %)	2 (0.1 %)		
Other	11 (0.9 %)	13 (1.0 %)	24 (0.91 %)		
Language spoken other than Hindi*n (%)					
Yes	817 (63.63 %)	937 (69.6 %)	1754 (66.66 %)	12.00	**<0.01**
No	460 (35.83 %)	395 (29.32 %)	855 (32.5 %)		
**Father's highest education***n (%)*					
Primary school	403 (31.4 %)	462 (34.3 %)	865 (32.9 %)	4.98	0.17
Secondary school	500 (38.94 %)	423 (31.4 %)	923 (35.1 %)		
University	104 (8.1 %)	101 (7.5 %)	205 (7.8 %)		
No formal education	25 (1.95 %)	36 (2.7 %)	61 (2.32 %)		
**Mother's highest education***n (%)*					
Primary school	529 (41.2 %)	529 (39.3 %)	980 (37.25 %)	5.4	0.15
Secondary school	169 (13.2 %)	169 (12.55 %)	343 (13.04 %)		
University	27 (2.0 %)	27 (2.0 %)	62 (2.4 %)		
No formal education	163 (12.1 %)	163 (12.1 %)	343 (13.04 %)		
**TV at home***n (%)*					
Yes	833 (64.9 %)	856 (63.55 %)	1689 (64.19 %)	0.62	0.62
No	436 (34.0 %)	467 (34.7 %)	903 (34.32 %)		
**Computer at home***n (%)*					
Yes	191 (14.9 %)	186 (13.81 %)	377 (14.33 %)	0.32	0.58
No	1064 (82.9 %)	1104 (82.0 %)	2168 (82.4 %)		
**Access to a mobile phone***n (%)*					
Yes	1194 (93.0 %)	1201 (89.2 %)	2395 (91.03 %)	6.25	**<0.01**
No	67 (5.22 %)	101 (7.5 %)	168 (6.38 %)		

*Note.* The cumulative percentages may not add up to 100 %, as some participants did not complete all questions

⁎ Most common language other than Hindi was Marwari, with 14 other languages indicated

Regarding comparisons on outcome variables at baseline (Table 3), the intervention group had significantly higher body esteem, positive affect, and body hair satisfaction, whilst the control group had significantly higher internalisation of appearance ideals and eating pathology (*p* < 0.05). Baseline measures were included as covariates in efficacy analyses.

**Table 3:** Unadjusted means and standard deviations for each outcome by condition at T2 and T3, with between-group comparisons (controlling for baseline differences in each outcome variable) and associated partial eta-squared effect sizes.

Outcome	Intervention *M (SD)*	Control *M (SD)*	*p* value	Partial eta-squared (ηp2) [95 % CI]
Body esteem				
T1	3.93 (0.60)	3.89 (0.60)	**<0.05**	-
T2	3.85 (0.62)	3.86 (0.66)	**.006**	0.003 (0.004, 0.284)
T3	3.83 (0.62)	3.70 (0.70)	**<0.001**	0.010 (0.007, 0.280)
Eating pathology				
T1	0.91 (0.71)	1.03 (0.77)	**<0.001**	-
T2	1.05 (0.89)	1.17 (0.92)	**.032**	0.002 (0.002, 0.267)
T3	1.23 (0.97)	1.41 (0.99)	**<0.001**	0.008 (0.008, 0.378)
Internalisation				
T1	2.99 (0.97)	3.09 (0.93)	**<0.05**	-
T2	1.95 (0.78)	2.79 (0.99)	**<0.001**	0.007 (0.007, 0.307)
T3	2.10 (0.95)	2.84 (0.98)	**<0.001**	0.130 (0.037, 0.260)
Life engagement				
T1	3.43 (0.53)	3.43 (0.55)	0.990	-
T2	3.50 (0.51)	3.47 (0.51)	0.118	0.001 (0.001, 0.438)
T3	3.41 (0.52)	3.26 (0.61)	**<0.001**^b^	0.005 (0.005, 0.344)
Positive affect				
T1	3.28 (0.99)	3.11 (1.00)	**<0.001**	-
T2	3.37 (1.04)	3.18 (1.02)	**<0.001**^a^	0.006 (0.006, 0.378)
T3	3.19 (1.01)	3.14 (1.00)	0.597	0.000 (0.000, 0.372)
Negative affect				
T1	2.33 (0.93)	2.27 (0.86)	0.120	-
T2	2.15 (0.90)	2.23 (0.88)	0.142	0.001 (.002, 0.371)
T3	2.16 (0.90)	2.29 (0.88)	0.119	0.001 (0.004, 0.383)
Endorsement of gender stereotypes				
T1	2.90 (0.81)	2.95 (0.88)	0.170	-
T2	2.67 (0.88)	2.74 (0.90)	**0.035**	0.002 (0.002, 0.333)
T3	2.68 (0.88)	2.82 (0.82)	**<0.001**	0.006 (0.006, 0.370)

Note: Significance threshold set at *p* < 0.05, indicated in bold.

a Significant differences were identified for girls only, with no significant differences identified between boys.

b Significant differences were identified for boys only, with no significant differences identified between girls.

Dropout rates were below 10 %, with maximum dropout of 9.42 % in the intervention group at T3. Since the preliminary power analyses accounted for attrition rates of 50 %, alpha = 0.05, and 1-beta = 0.80 and Cohen's *d* = 0.2, it was concluded that the analyses were sufficiently powered despite the dropouts. Missing data was due to missed items and student absences at post-intervention. Missing data did not vary by gender (*p* = 0.86). Little's MCAR test indicated that missingness was consistent with data being missing completely at random between T1 and T2 (*p* = 0.065), but not between T1 and T3 (*p* = 0.034). However, independent sample *t*-tests confirmed that missing data for the primary outcome measure (body esteem) at both T2 and T3 was not dependent on baseline values of trait body satisfaction (*t*(2526)= –1.158, *p* = 0.25 at T2; t(2526)= –0.001, *p* = 0.999 at T3).

### Efficacy outcomes

Table 3 displays the unadjusted mean values, standard deviations, between-group comparisons and associated effect sizes for primary and secondary outcomes, whilst Tables S4–S8 include the percentage values for the exploratory measures, at all three time-points and across both conditions.

With regard to the primary outcome, findings unexpectedly indicated a worsening of body esteem across time in both conditions. However, intervention participants experienced significantly higher body esteem at post-intervention compared with control participants (η_p_^2^ = 0.003), with effects maintained at follow-up (η_p_^2^ = 0.010).

A similar surprising trend was observed for eating pathology, whereby all participants (irrespective of their condition) appeared to report increases in eating pathology across time. Nonetheless, intervention participants reported significantly lower eating pathology at both post-intervention (η_p_^2^ = 0.002) and follow-up (eating pathology, η_p_^2^ = 0.008). Regarding other secondary outcomes, trends were in the expected direction, whereby improvements were indicated among intervention participants over time relative to control participants. Intervention participants reported significantly lower internalisation at both post-intervention (η_p_^2^ = 0.007) and follow-up (η_p_^2^ = 0.130). Whilst there were no significant differences in life engagement at post-intervention, intervention participants reported significantly higher life engagement at follow-up (η_p_^2^ = 0.005). Regarding affect, whilst intervention students experienced significantly higher positive affect at post-intervention (η_p_^2^ = 0.006), this was not maintained at follow-up, and there were no significant differences in negative affect at any point.

Regarding exploratory outcomes, trends were as anticipated, whereby intervention participants appeared to improve over time relative to control participants. Analyses revealed that intervention participants experienced significantly lower endorsement of gender stereotypes at both post-intervention (η_p_^2^ = 0.002) and follow-up (η_p_^2^ = 0.006). For the measures assessing skin shade satisfaction, whilst the skin shade rating scale identified significantly higher skin shade satisfaction among both genders at post-intervention (girls, *p* = 0.003; Nagelkerke's R^2^ = 0.034; boys *p* =< 0.001; Nagelkerke's R^2^ = 0.032) and follow-up (girls, *p* =< 0.001, Nagelkerke's R^2^ = 0.036; boys, *p* = 0.002; Nagelkerke's R^2^ = 0.025), the single-item scale detected significantly higher skin shade satisfaction for boys only, and just at follow-up (*p* = 0.006; Nagelkerke's R^2^ = 0.012). Similarly, there was significantly higher body hair satisfaction for intervention boys only (i.e., not among girls) and solely at follow-up (*p* =< 0.001; Nagelkerke's R^2^ = 0.022), whilst significantly lower appearance-based teasing was also only identified among boys, and at post-intervention alone (*p* =< 0.001; Nagelkerke's R^2^ = 0.026). There were no significant between-group differences at any point for body size satisfaction in either gender.

### Fidelity outcomes

Good inter-rater reliability (ICC values > 0.7) was achieved for all items, including the domains of perceived facilitator competency (*M* = 5.56) and perceived facilitator adherence (*M* = 5.18). Adherence, as indicated by the proportion of key questions and actions completed by teachers, ranged between 66 % and 78 % across the six sessions (*M* = 75 %). Each session lasted between 36 and 43 min (*M* = 41 min), approximating the recommended timing (45 min per session).

### Acceptability outcomes

Quantitative findings indicated high intervention acceptability among both students (> 75 %) and teachers (> 84 %; Table S1). Qualitative findings also revealed positive feedback on the sessions (Table S2), such as both teachers and students expressing that the comics were relatable and should be rolled out at scale. Teachers felt the intervention was relevant, language was accessible, and the use of comics to teach was innovative. Teachers attributed their confidence in intervention delivery to the teacher guide and the training they received. However, both teachers and students highlighted that the activities could be made easier (e.g., by using multiple-choice format). Overall, both teachers and students felt that the intervention should be rolled out at scale among Indian adolescents.

### Validity outcomes

Regarding key learnings, 1051 responses (95.5 % of intervention group) included at least one desired response (Table S3). Of those, 843 (77 %) responses directly mapped onto at least one of the key learning outcomes from the six sessions. Some responses reflected broader messaging across the intervention, such as focusing on qualities and not appearance (190 responses, 17 %), not worrying about looks (156 responses, 14.23 %), and not judging people based on their appearance (20 responses, 2 %). Overall, these findings support the validity of the quantitative efficacy findings.

## Discussion

This study aimed to evaluate the efficacy of a mixed-gender comic-based body image intervention among adolescents in semi-rural Indian schools. Unexpectedly, both the primary outcome of body esteem and secondary outcome of eating pathology worsened among students who undertook the intervention. Nonetheless, body esteem was significantly higher among intervention students (relative to those in the control arm) at post-intervention, which was maintained three months later. These findings are promising, given that this is the first body image intervention to be evaluated among adolescents in semi-rural India, and extends similar findings among an urban sample of Indian adolescents (Lewis-Smith et al., 2023). The effect size relating to body esteem was small (at follow-up), reflecting the previous evaluation of a mixed-gender school-based body image intervention in India (Lewis-Smith et al., 2023) and other universal trials globally (e.g., Buerger et al., 2019; Diedrichs et al., 2021; Yager et al., 2013b). Nonetheless, smaller effect sizes are expected in universal interventions (Watson et al., 2016) and can convert into substantial public mental health benefits when disseminated at scale (Patel et al., 2007).

The significant effects for most secondary and exploratory outcomes were also encouraging, given the inconsistency of effects for risk factors of body dissatisfaction and related outcomes across prior school-based intervention evaluations (Yager et al., 2013b). The present effects mirrored those of the earlier Indian study, including significantly lower levels of eating pathology and internalisation of appearance ideals among students in the intervention arm (relative to students in the control arm), which were maintained three months later (Lewis-Smith et al., 2023). However, it also indicated maintained benefits to other novel outcomes, including skin shade satisfaction and reduced endorsement of gender stereotypes. These are encouraging findings, given that skin shade is a salient body image concern in India, leading to the use of harmful skin lightening products, and with colourism perpetuating social inequalities (Kukreja, 2021; Peltzer et al., 2016; Shroff et al., 2018). Similarly, gender stereotyping is a significant issue in more rural and lower socio-economic regions of India (Mohan, 2017; Waghachavare, Dhumale, & Kadam, 2021) and is associated with poorer health outcomes (McCarthy, Mehta, & Haberland, 2018; Singh, Bloom, & Brodish, 2015), but interventions are lacking. Therefore, the present intervention offers hope in being able to target common social justice issues that are adversely associated with health among adolescents in India.

The absence of effects for girls in relation to body image-related life engagement and body hair satisfaction warrants consideration. First, life engagement, measured by the BILD-Q, includes activities (e.g., “physical activity or sport”) which could be less applicable to girls in semi-rural India. Additionally, this measure implies a choice to participate in these activities, whereas girls are likely to have less choice and might require parental supervision (Batra & Reio Jr, 2016; Jayachandran, 2015). Considering the absence of effects among girls for body hair satisfaction, this may relate to pubertal timing. Indeed, the growth of body hair accompanies puberty, and tends to be “complete” by age 14 in low- and middle- income countries (Moodie et al., 2020). Undernutrition is common in rural regions of India and can delay puberty (Soliman, De Sanctis, & Elalaily, 2014), meaning that adolescents in the present sample may have had little body hair. Therefore, boys may have felt dissatisfied with their limited body hair, which is traditionally associated with masculinity (Synnott, 1987), whilst girls did not have enough body hair to experience any dissatisfaction that has previously been noted (Phadke, 2017).

The absence of effects or maintained improvements in appearance-based teasing, positive affect, and negative affect support previous evaluations in India and beyond (Diedrichs et al., 2021, Lewis-Smith et al., 2023; Yager et al., 2013^b^). Given that Indian families tend to comment on relatives’ appearance (Dhillon & Dhawan, 2011), students could still have been experiencing negative comments and teasing at home, despite reductions from peers in class, with boys returning to their usual “banter” and teasing among one another after shortly after their reductions at post-intervention (Doley, McLean, Griffiths, & Yager, 2021). Regarding positive and negative affect, these are broad psychological constructs that are likely to be influenced by external factors beyond body image, and thus are likely to return to baseline levels. Further, items representing negative emotions may have impeded an honest response, due to stigma associated with experiencing poor mental health in India, particularly in more rural regions (Kudva et al., 2020). Finally, the absence of significant effects for body size satisfaction also warrant consideration. These findings suggest that weight and size may be less relevant to the body image of adolescents in rural and lower-socioeconomic Rajasthan, where there is a “dual burden” of underweight and overweight, compared with more urban settings, like Delhi (Ramadass, Gupta, & Nongkynrih, 2017; Singh & Amita, 2021). Irrespective, further research exploring the nature of body image concerns is needed among adolescents in more rural regions of India.

Whilst findings indicate significantly higher body esteem and lower eating pathology among adolescents in the intervention condition relative to the control condition, the suggested trend for the worsening of these outcomes among *all* young people warrants discussion. Whilst these findings seem counterintuitive, they could be interpreted within the broader context of external social factors. First, a religious festival involving fasting of food was taking place during the period within which post-intervention data collection occurred, and previous investigations among young adults and adolescents suggest that religious fasting can trigger eating pathology and body image concerns (Akgül et al., 2014; Hasan et al., 2021; Vaidyanathan et al., 2019). Second, students were undertaking school exams during the period within which follow-up data collection took place and might have consequently been experiencing a higher level of stress than usual. Stress has been associated with body image concerns and eating pathology among adolescents, with the latter partially attributed to emotion-oriented coping (Henderson et al., 2022; Hill., 2018; Murray et al., 2013). Collectively, this suggests that external pressures could have accounted for the worsening of body esteem and eating pathology in both conditions, with the intervention acting as protective factor for adolescents. Nonetheless, these interpretations are speculative and should be treated with caution.

Whilst constituting the first teacher-delivered school-based body image intervention to be evaluated among adolescents in India, the research has limitations. First, it was not feasible to measure body mass index or pubertal status. However, given that approximately a third of adolescents in such regions are underweight, with nutritional deficiency widespread (Singh & Amita, 2021), appearance-related concerns for weight and other aspects relating to more advanced puberty (e.g., body hair) may be irrelevant to this group (Weinberger, Kersting, Riedel-Heller, & Luck-Sikorski, 2016). Second, teachers and data collectors were not blinded during the trial due to the psychosocial nature of the intervention and the absence of an active control. However, statisticians and authors were blinded to allocated conditions. Third, reflecting previous evaluations of Indian teacher-delivered mental health interventions (e.g., Leventhal et al., 2018), intervention fidelity was sub-optimal. However, this is not surprising, given that Indian teachers in rural government schools are understaffed and overworked (Business Today, 2021; Indian Development Review, 2021). Therefore, the present findings are arguably promising, given the realistic likelihood that teachers may not have capacity to follow the intervention guide closely. Nonetheless, unlike most prior evaluations of Indian school-based mental health interventions (e.g., Singla, Shinde, Patton, & Patel, 2021; Tidwell et al., 2020), a strength of the present research is that fidelity *was* measured. Regarding other strengths of the research, these include the step-based and co-creation approach (with UNICEF, BBC Media Action, DSEP) to develop and evaluate the acceptability of the comic-based intervention (Ahuja et al., 2022), in addition to the use of rigorously validated Hindi psychosocial measures, and finally, the fully powered large sample size across 41 schools.

Although the present findings highlight schools as a fruitful site for the delivery of mental health interventions to Indian adolescents, research suggests that up to 41 % of girls in both urban and rural India do not attend school when menstruating, with some dropping out of education entirely (Vashisht et al., 2018; Yaliwal et al., 2020). Whilst policy- and system- level initiatives are being pursued to address this (Kishore et al., 2022), alternative avenues by which adolescent girls can be reached should be considered. One such route is via community groups, with recent research indicating promise for a body image programme delivered to Indian adolescent girls as part of the World Association of Girl Guides and Girl Scouts (the largest voluntary movement dedicated to girls and young women in the world; Paraskeva et al., 2024). Nonetheless, we recognise that any community-based intervention can be costly, and thus recommend that researchers consider settings that require fewer resources. One such contender is the digital environment, given that increasing numbers of Indian adolescents, including those living in more rural regions, have access to a smartphone. Indeed, at least half of all adolescents living in rural India are suggested to have access to a smartphone and engage regularly with online videos, games, and social media (Statista, 2022, 2023). Recent research has demonstrated the benefits of body image interventions that have been embedded within the digital media landscape for adolescents in other middle- and low- income countries (e.g., Garbett et al., 2023; Matheson et al., 2023), thus suggesting a potential avenue by which thousands of Indian adolescents could receive body image support using minimal resources.

## Conclusion

This is the first study to evaluate a teacher-delivered and culturally appropriate school-based body image intervention among adolescents in semi-rural India. The maintained benefits relative to the control group for the primary outcome of body esteem, in addition to eating pathology, skin shade and body hair satisfaction, internalisation of appearance ideals, life engagement, and endorsement of gender stereotypes, are promising. This responds to calls for scalable mental health interventions that can be delivered by non-specialist professionals in low- and middle- income countries, and particularly in more rural areas (Barbui et al., 2020; De Sousa et al., 2020). It also demonstrates the use of comics as a robust intervention for promoting learning and better mental health among adolescents (Sinha, Patel, Kim, MacCorkle, & Watkins, 2011). The present intervention will be scaled up and disseminated by UNICEF across eight states of India, consequently helping to address the mental health needs of adolescents in this country.

## Declaration of competing interest

HLS and PD are independent consultants to the Dove Self-Esteem Project, and PD was on the Dove Self-Esteem Project Global Advisory Board from 2013–2016. The authors declare that they have no competing interests.

## Appendix Supplementary materials

Image, application 1

Image, application 2

Image, application 3

Image, application 4

Image, application 5

Image, application 6

Image, application 7

Image, application 8

Image, application 9

## Footnotes

1 Whilst colourism in India has historical and cultural roots in colonialism and the Indian caste system (Dixon & Telles, 2017; Kullrich, 2022), it is reinforced in the modern day by the media (e.g., Bollywood industry), advertisement industry, beauty industry, and Indian marriage market (Kukreja, 2021; Mitchell, 2020; Negi et al., 2024).

2 Most classes did not have time to engage in the second activity. Only one or two classes started this activity for each respective session, and often did not finish it. This was randomly distributed across different classes (i.e., it was not the same class that started the second activity each session).

3 Students this age begin to study specific subjects, including sciences, mathematics, arts, social sciences, and humanities. Whilst schools in urban India are becoming increasingly more interactive and encouraging of critical thinking and experiential learning, schools in more rural areas still tend to adopt the more traditional and didactic Guru-Shishya teaching model, which is based on the premise of a teacher (guru) transferring knowledge to a student (shishya; Government of India, 2020).

4 The validation processes and findings of these measures are reported in other manuscripts that are currently in preparation.

5 Since publication of the original protocol (Lewis-Smith et al., 2022), the decision was made to add two image-based outcome measures of skin shade satisfaction and body size satisfaction to complement other text-based measures. This was informed by further discussions with stakeholders regarding concerns about students’ low literacy levels.
