# Supplementary material for: A comic-based body image intervention for adolescents in semi-rural Indian schools: A randomised controlled trial
Source: Int J Clin Health Psychol. 2025 Jan 26;25(1):100546. doi: 10.1016/j.ijchp.2025.100546 (PMC11795790; doi:10.1016/j.ijchp.2025.100546)
Supplement: Supplementary file 1 [file mmc1.docx]

| Table S1*. Quantitative findings relating to student and facilitator acceptability of the intervention | | | |
| --- | --- | --- | --- |
| Student Question | Percentage of participants (%) N = 1347 | | |
|  | Agree | Neutral | Disagree |
| I enjoyed the sessions | 78.1 | 6.5 | 15.4 |
| The sessions helped me feel better about myself | 75.4 | 9.3 | 15.4 |
| I understood what was being taught in the sessions | 81.6 | 8.7 | 9.7 |
| I felt comfortable taking part in the sessions | 76.7 | 10.5 | 12.9 |
| The sessions were taught well by the teacher | 83.3 | 7.9 | 8.8 |
| It is important for young people to take part in sessions like these that improve body confidence | 76.0 | 11.8 | 12.1 |
| Facilitator Question | Facilitator Response (%) N = 50 | | |
|  | Yes (%) | Neutral (%) | No (%) |
| Did you enjoy delivering the sessions? | 96 | 4 | - |
| Did students appear to enjoy the sessions? | 86 | 12 | 2 |
| How confident did you feel in delivering the session | 96 | 4 | - |
| Do you think the sessions helped adolescents feel good about themselves? | 90 | 8 | 2 |
| Do you think the sessions helped adolescents with their body confidence? | 88 | 12 | - |
| Did students display understanding of the key messages of all the sessions? | 86 | 14 | - |
| Did students appear engaged in the topics and activities? | 90 | 8 | 2 |
| Do you feel you achieved the learning objectives of the sessions? | 84 | 16 | - |
| Was the Teacher Guide clear? | 90 | 8 | 2 |
| How useful did you find the resources for the body image programme? (i.e., teacher guide, training, slides) | 86 | 10 | 4 |

**Note*. The cumulative percentages may not add up to 100%, as some participants did not complete all questions.
