# Supplementary material for: A comic-based body image intervention for adolescents in semi-rural Indian schools: A randomised controlled trial
Source: Int J Clin Health Psychol. 2025 Jan 26;25(1):100546. doi: 10.1016/j.ijchp.2025.100546 (PMC11795790; doi:10.1016/j.ijchp.2025.100546)
Supplement: Supplementary file 2 [file mmc2.docx]

Table S2. Qualitative findings from the teacher and student focus groups

| Topic | Students’ Feedback | Teachers’ Feedback |
| --- | --- | --- |
| **Relevance and prior learning of body image** | | |
| Prior training or knowledge on body image (or gender stereotypes) | - Students had not received prior learning on body image. - Some students had previously learned about gender equality at school, but not within the context of body image. | - Teachers had no prior knowledge or training relating to body image concerns or its risk factors. |
| Prevalence of body image concerns at school | - Most students highlighted that appearance concerns relating to weight, shape, and skin colour/texture, and appearance-related teasing were common. | - All teachers acknowledged that body image concerns (particularly expressed via appearance comparisons and body talk) and gender stereotypes were prevalent among their students. - Teachers reflected on the impact of media on appearance ideals and discussed how students were influenced by media messages, including on social media. For example, they wanted to go to the gym, and change their appearance. - Some teachers felt that body image concerns were more common among students in class 8 rather than in class 6 and 7. |
| **Training and preparation for the programme** | | |
| Experience of teacher training | - *Not relevant for students* | - All teachers acknowledged that the training in a group was helpful in delivering the comics as it was a new topic. - Teachers highlighted that the training helped them facilitate, rather than deliver, the sessions, and to ensure sensitivity when doing so. |
| Process of preparation for sessions | - *Not relevant for students* | - Teachers found it easy to prepare for the sessions once they understood all the concepts from the training. - The preparation took as long as it normally took them to prepare for their usual lessons. |
| **Feedback on intervention material** | | |
| Interest in comics and engagement with characters | - All students found the comics novel, related to the characters, and enjoyed reading them. They were particularly enthused about Comic 1 on gender stereotypes, Comic 3 about media messages, and Comic 5 on body talk, due to the relevance of these concerns in their lives. - Nearly everyone found the comics relevant to the Indian context. | - Teachers felt that students really enjoyed reading and engaging with the comics. - *Teachers had little discussions about students’ engagement with the characters in the comics.* |
| Learnings and comprehension of the comics | - All students were able to recall the overall key learnings and themes from the comics (e.g., body talk is bad we shouldn’t tease), but struggled to recall the nuanced key learnings (e.g., the societal impact of gender stereotypes, appearance ideals, and comparisons). - While all students agreed that the comics were age-appropriate, some felt that these could be taught to younger students, due to their belief that these issues need to be addressed from an earlier age. - All students thought that these comics should be taught to other students their age. Some of them shared the comics with their friends and family, especially as they thought ‘gender stereotypes’ and ‘body talk’ should be taught to everyone. | - Some teachers highlighted that they had to simplify the content to explain it to younger students. These students also struggled with understanding the key learnings, especially as literacy levels are low in rural areas. - Some teachers struggled to keep to the recommended timings. - Teachers reported that while the language of the comics was easy, students needed support to understand new terms (e.g., stereotypes, body image), and thus had to use the local language and comic imagery to facilitate understanding. |
| Engagement with the activities | - Students enjoyed completing the activities but found the ‘match the following’ format difficult. - Students in higher grades liked the sentence completion activity (where they had to think of a response), whereas students in lower grades found them challenging. - Most students felt the teachers’ support was necessary to complete the activities, generally. - Overall, while activities were challenging, students enjoyed that they were different from those they usually did in class. - There was mixed opinion on whether the activities helped them learn key learnings in a better way. However, some students did agree that the activities targeted key learnings. | - Teachers reported that engagement with activities was low for students from classes 6 and 7, as they found the activities challenging and confusing. Students in higher grades were able to complete the activities. - Teachers highlighted that student engagement with activities was high across both boys and girls. All students enjoyed the lessons and waited for the next set of comics. However, teachers felt that generally, girls were more engaged and excited than boys as they were more influenced by media-propagated appearance ideals. |
| Programme delivery | - The students felt that the teachers delivered the sessions well, and this helped them better understand the comics, activities, and difficult concepts (e.g., gender stereotypes, body talk). | - All teachers enjoyed delivering the body image programme. - Some teachers initially found it challenging to be recorded and found the first session difficult. However, they became more comfortable as the sessions went on, and overall, teachers had no issues with being recorded. - All teachers felt that the comics should be taught in mixed-gender groups. - External factors such as noise, or other students playing outside often distracted students, but teachers worked to manage the situation. - Teachers also discussed the challenges in scheduling the comic lessons, as there was a lot of pressure from school authorities to complete the syllabus by a recommended date. - Teachers also struggled with lack of support from other teachers, who were not aware of the body image lessons, and therefore, would be impatient if the sessions were running over time, creating a lot of pressure. |
| Benefits of the programme | - The comics helped nearly all students to stop focusing on appearance (e.g., stop appearance-based comments) and focus on their own and others’ qualities instead. It also increased their self-confidence. Behavioural changes were also discussed by students, e.g., they stopped putting gel in their hair. They also noticed changes in their peers, e.g., students stopped teasing each other. - All students felt everyone could benefit from learning about these issues. All students discussed the comics with their siblings, parents, and friends. - All students felt it was very important that all students and young people learned about these issues – specifically about teasing and gender stereotyping. | - Teachers acknowledged that the comics really helped students understand body image concerns and their impact. - The comics helped students better understand concepts related to media messages and teasing. - Teachers observed a change in students’ behaviour and thought process (change in their appearance, stopped worrying about their hair styles etc), though hard to say if they will be long-term. |
| Experience of using Teacher Guide | - *Not relevant for students* | - Most teachers found the teacher guide helpful while delivering the sessions. However, some teachers did not read the entire guide, due to its length and limited time. They only briefly read the instructions and main points necessary to deliver the sessions (right before they had to deliver the session). - All teachers found the language and format of the teacher guide easy to understand and follow while delivering the sessions. - The teachers mentioned that the bold text, and the summary of each comic and key learnings were helpful in delivering the sessions. - The teachers also mentioned that they found it challenging to stick to the allocated time in the teacher guide and would have preferred if they could instead have focussed entirely on the students, without worrying about specific timings for each section. |
