# Supplementary material for: A comic-based body image intervention for adolescents in semi-rural Indian schools: A randomised controlled trial
Source: Int J Clin Health Psychol. 2025 Jan 26;25(1):100546. doi: 10.1016/j.ijchp.2025.100546 (PMC11795790; doi:10.1016/j.ijchp.2025.100546)
Supplement: Supplementary file 3 [file mmc3.docx]

Table S3. Themes and illustrative quotes regarding student learnings from the intervention

| Comic 1: Gender Stereotypes (n = 416) |
| --- |
| Girls can do everything that boys can |
| We should not treat boys and girls differently because of their gender |
| Comic 2: Appearance Ideals (n = 267) |
| We should not try to become/look like the models on TV and in movies |
| We should not aspire to achieve appearance ideals |
| Comic 3: Media Messages (n =237) |
| What we see in TV and movies is not real |
| The images on TV, and movies are edited to sell products |
| Comic 4: Appearance Comparisons (n = 486) |
| We should not compare our appearance to others |
| We should not compare ourselves to anyone else |
| Comic 5: Body Talk (n = 139) |
| We should not comment on anyone’s appearance, whether good or bad We should not do body talk |
| We are all unique and should embrace this (n = 190) |
| We should not try to look like anyone else |
| We are good just as we are, and everyone is unique |
| We should not focus on or worry about the way we look (n = 156) |
| We should not worry about our looks and appearance  We should not run after looks (but instead focus on qualities) |
| We should not judge or treat people in a certain way because of how they look (n =20) |
| We should not treat people differently based on their looks  We should not discriminate between people because of their skin colour or their body size |
|  |
