# Supplementary material for: A comic-based body image intervention for adolescents in semi-rural Indian schools: A randomised controlled trial
Source: Int J Clin Health Psychol. 2025 Jan 26;25(1):100546. doi: 10.1016/j.ijchp.2025.100546 (PMC11795790; doi:10.1016/j.ijchp.2025.100546)
Supplement: Supplementary file 4 [file mmc4.docx]

S4. Frequency and percentages for skin colour satisfaction (Likert scale) at T1, T2, and T3

|  | | Girls (n = 1287) | | | *x^2^* value | *p* value | Boys (n = 1270) | | | | *x^2^* value | *p* value |
| --- | --- | --- | --- | --- | --- | --- | --- | --- | --- | --- | --- | --- |
|  | | Intervention  (n = 642) | | Control  (n = 645) |  |  | Intervention  (n = 673) | | Control  (n = 597) | |  |  |
| **T1** | | | | | | | | | | |  |  |
| *Very dissatisfied* | | 135 (21.0%) | | 132 (20.5%) |  |  | 155 (23.0%) | | 108 (18.1%) | |  |  |
| *Mostly dissatisfied* | | 80 (12.5%) | | 91 (14.1%) |  |  | 97 (14.4%) | | 110 (18.4%) | |  |  |
| *Neither dissatisfied nor satisfied* | | 115 (17.9%) | | 108 (16.7%) |  |  | 131 (19.5%) | | 103 (17.3%) | |  |  |
| *Mostly satisfied* | | 96 (15.0%) | | 101 (15.7%) |  |  | 109 (16.2%) | | 103 (17.3%) | |  |  |
| *Very satisfied* | | 216 (33.6%) | | 213 (33.0%) |  |  | 181 (26.9%) | | 173 (29.0%) | |  |  |
| **T2** | |  | |  | 0.004 | 0.950 |  | |  | | 1.737 | .188 |
| *Very dissatisfied* | | 99 (15.8%) | | 89 (14.6%) |  |  | 80 (12.0%) | | 76 (13.3%) | |  |  |
| *Mostly dissatisfied* | | 67 (10.7%) | | 49 (8.1%) |  |  | 68 (10.2%) | | 65 (11.3%) | |  |  |
| *Neither dissatisfied nor satisfied* | | 82 (13.1%) | | 105 (17.3%) |  |  | 109 (16.4%) | | 96 (16.8%) | |  |  |
| *Mostly satisfied* | | 128 (20.4%) | | 135 (22.2%) |  |  | 154 (23.2%) | | 137 (23.9%) | |  |  |
| *Very satisfied* | | 250 (39.9%) | | 230 (37.8%) |  |  | 253 (38.1%) | | 199 (34.7%) | |  |  |
| **T3** |  | |  | | 0.706 | .401 | |  | |  | **7.445** | **.006** |
| *Very dissatisfied* | | 130 (20.8%) | | 88 (14.6%) |  |  | 114 (17.3%) | | 82 (14.6%) | |  |  |
| *Mostly dissatisfied* | | 72 (11.5%) | | 78 (13.0%) |  |  | 71 (10.8%) | | 94 (16.7%) | |  |  |
| *Neither dissatisfied nor satisfied* | | 123 (19.7%) | | 136 (22.6%) |  |  | 110 (16.7%) | | 141 (25.1%) | |  |  |
| *Mostly satisfied* | | 104 (16.6%) | | 123 (20.5%) |  |  | 139 (21.1%) | | 100 (17.8%) | |  |  |
| *Very satisfied* | | 196 (31.4%) | | 176 (29.3%) |  |  | 225 (34.1%) | | 145 (25.8%) | |  |  |

Note: Chi-square and p-value for between groups effect in an ordinal logistic regression with baseline as a covariate.
