# Supplementary material for: A comic-based body image intervention for adolescents in semi-rural Indian schools: A randomised controlled trial
Source: Int J Clin Health Psychol. 2025 Jan 26;25(1):100546. doi: 10.1016/j.ijchp.2025.100546 (PMC11795790; doi:10.1016/j.ijchp.2025.100546)
Supplement: Supplementary file 5 [file mmc5.docx]

S5. Frequency and percentages for skin colour satisfaction (skin shade rating scale) at T1, T2, and T3

|  | Girls (n = 1281) | | | | *x^2^* value | *p* value | | Boys (n = 1257) | | | | *x^2^* value | | *p* value |
| --- | --- | --- | --- | --- | --- | --- | --- | --- | --- | --- | --- | --- | --- | --- |
|  | Intervention  (n = 642) | | Control  (n = 639) | |  | |  | Intervention  (n = 666) | Control  (n = 591) | | |  | |  |
| **T1** | | | | | | | | | | | |  | |  |
| *0 (Very satisfied)* | 203 (31.6%) | | 205 (32.1%) | |  | |  | 243 (36.5%) | 179 (30.3%) | | |  | |  |
| *1* | 306 (47.7%) | | 314 (49.1%) | |  | |  | 268 (40.2%) | 222 (37.6%) | | |  | |  |
| *2* | 81 (12.6%) | | 77 (12.1%) | |  | |  | 88 (13.2%) | 118 (20.0%) | | |  | |  |
| *3* | 35 (5.5%) | | 30 (4.7%) | |  | |  | 43 (6.5%) | 53 (9.0%) | | |  | |  |
| *4* | 6 (0.9%) | | 8 (1.3%) | |  | |  | 10 (1.5%) | 8 (1.4%) | | |  | |  |
| *5* | 3 (0.5%) | | 2 (0.3%) | |  | |  | 6 (0.9%) | 8 (1.4%) | | |  | |  |
| *6* | 6 (0.9%) | | 1 (0.2%) | |  | |  | 5 (0.8%) | 2 (0.3%) | | |  | |  |
| *7* | 2 (0.3%) | | 2 (0.3%) | |  | |  | 2 (0.3%) | 1 (0.2%) | | |  | |  |
| *8 (Very dissatisfied)* | - | | - | |  | |  | 1 (0.2%) | - | | |  | |  |
| **T2** |  | |  | | **8.747** | | **.003** |  |  | | | **12.945** | | **<.001** |
| *0 (Very satisfied)* | 266 (42.4%) | | 210 (34.5%) | |  | |  | 283 (42.6%) | 180 (31.4%) | | |  | |  |
| *1* | 242 (38.6%) | | 265 (43.6%) | |  | |  | 232 (34.9%) | 241 (42.1%) | | |  | |  |
| *2* | 76 (12.1%) | | 72 (11.8%) | |  | |  | 101 (15.2%) | 93 (16.2%) | | |  | |  |
| *3* | 30 (4.8%) | | 55 (9.0%) | |  | |  | 40 (6.0%) | 45 (7.9%) | | |  | |  |
| *4* | 9 (1.4%) | | 3 (0.5%) | |  | |  | 8 (1.2%) | 6 (1.0%) | | |  | |  |
| *5* | 4 (0.6%) | | 2 (0.3%) | |  | |  | 1 (0.2%) | 5 (0.9%) | | |  | |  |
| *6* | - | | 1 (0.2%) | |  | |  | - | 1 (0.2%) | | |  | |  |
| *7* | - | | - | |  | |  | - | 1 (0.2%) | | |  | |  |
| *8 (Very dissatisfied)* | - | | - | |  | |  | - | - | | |  | |  |
| **T3** | |  | |  | **19.385** | | **<.001** | | |  |  | | **9.639** | **.002** |
| *0 (Very satisfied)* | 280 (44.8%) | | 222 (36.9%) | |  | |  | 286 (43.5%) | 195 (34.8%) | | |  | |  |
| *1* | 241 (38.6%) | | 218 (36.3%) | |  | |  | 242 (36.8%) | 234 (41.7%) | | |  | |  |
| *2* | 79 (12.6%) | | 114 (19.0%) | |  | |  | 93 (14.1%) | 96 (17.1%) | | |  | |  |
| *3* | 20 (3.2%) | | 29 (4.8%) | |  | |  | 32 (4.9%) | 27 (4.8%) | | |  | |  |
| *4* | 5 (0.8%) | | 12 (2.0%) | |  | |  | 3 (0.5%) | 6 (1.1%) | | |  | |  |
| *5* | - | | 4 (0.7%) | |  | |  | 2 (0.3%) | 1 (0.2%) | | |  | |  |
| *6* | - | | 2 (0.3%) | |  | |  | - | 1 (0.2%) | | |  | |  |
| *7* | - | | - | |  | |  | - | 1 (0.2%) | | |  | |  |
| *8 (Very dissatisfied)* | - | | - | |  | |  | - | - | | |  | |  |

Note: Chi-square and p-value for between groups effect in an ordinal logistic regression with baseline as a covariate.
