# Supplementary material for: A comic-based body image intervention for adolescents in semi-rural Indian schools: A randomised controlled trial
Source: Int J Clin Health Psychol. 2025 Jan 26;25(1):100546. doi: 10.1016/j.ijchp.2025.100546 (PMC11795790; doi:10.1016/j.ijchp.2025.100546)
Supplement: Supplementary file 6 [file mmc6.docx]

S6. Frequency and percentages for body hair satisfaction at T1, T2, and T3

|  | | | Girls (n = 1266) | | *x^2^* value | *p* value | Boys (n = 1261) | | | *x^2^* value | *p* value |
| --- | --- | --- | --- | --- | --- | --- | --- | --- | --- | --- | --- |
|  | | | Intervention  (n = 633) | Control  (n = 633) |  |  | Intervention  (n = 671) | Control  (n = 590) | |  |  |
| **T1** | | | | | | | | | |  |  |
| *Very dissatisfied* | | | 141 (22.3%) | 174 (27.5%) |  |  | 122 (18.2%) | 148 (25.1%) | |  |  |
| *Mostly dissatisfied* | | | 81 (12.8%) | 89 (14.1%) |  |  | 93 (13.9%) | 74 (12.5%) | |  |  |
| *Neither dissatisfied nor satisfied* | | | 137 (21.6%) | 117 (18.5%) |  |  | 142 (21.2%) | 91 (15.4%) | |  |  |
| *Mostly satisfied* | | | 98 (15.5%) | 91 (14.4%) |  |  | 112 (16.7%) | 102 (17.3%) | |  |  |
| *Very satisfied* | | | 176 (27.8%) | 162 (25.6%) |  |  | 202 (30.1%) | 175 (29.7%) | |  |  |
| **T2** | | |  |  | 2.850 | .091 |  |  | | 2.804 | .094 |
| *Very dissatisfied* | | | 125 (20.0%) | 142 (23.6%) |  |  | 99 (15.0%) | 104 (18.2%) | |  |  |
| *Mostly dissatisfied* | | | 70 (11.2%) | 44 (7.3%) |  |  | 63 (9.5%) | 62 (10.9%) | |  |  |
| *Neither dissatisfied nor satisfied* | | | 121 (19.4%) | 153 (25.4%) |  |  | 139 (21.0%) | 122 (21.4%) | |  |  |
| *Mostly satisfied* | | | 119 (19.1%) | 103 (17.1%) |  |  | 132 (20.0%) | 111 (19.4%) | |  |  |
| *Very satisfied* | | | 189 (30.3%) | 160 (26.6%) |  |  | 228 (34.5%) | 172 (30.1%) | |  |  |
| **T3** |  |  | | | 1.048 | .306 |  | |  | **13.746** | **<.001** |
| *Very dissatisfied* | | | 152 (24.4%) | 116 (19.3%) |  |  | 106 (16.1%) | 112 (19.9%) | |  |  |
| *Mostly dissatisfied* | | | 55 (8.8%) | 64 (10.7%) |  |  | 67 (10.2%) | 102 (18.1%) | |  |  |
| *Neither dissatisfied nor satisfied* | | | 142 (22.8%) | 162 (27.0%) |  |  | 147 (22.3%) | 122 (21.7%) | |  |  |
| *Mostly satisfied* | | | 109 (17.5%) | 89 (14.8%) |  |  | 132 (20.0%) | 90 (16.0%) | |  |  |
| *Very satisfied* | | | 166 (26.6%) | 169 (28.2%) |  |  | 207 (31.4%) | 136 (24.2%) | |  |  |

Note: Chi-square and p-value for between groups effect in an ordinal logistic regression with baseline as a covariate.
