# Supplementary material for: A comic-based body image intervention for adolescents in semi-rural Indian schools: A randomised controlled trial
Source: Int J Clin Health Psychol. 2025 Jan 26;25(1):100546. doi: 10.1016/j.ijchp.2025.100546 (PMC11795790; doi:10.1016/j.ijchp.2025.100546)
Supplement: Supplementary file 7 [file mmc7.docx]

S7. Frequency and percentages for appearance-based teasing at T1, T2, and T3

|  | Girls (n = 1284) | | | *x^2^* value | *p* value | Boys (n = 1264) | | | | *x^2^* value | *p* value |
| --- | --- | --- | --- | --- | --- | --- | --- | --- | --- | --- | --- |
|  | Intervention  (n = 644) | | Control  (n = 640) |  |  | Intervention  (n = 640) | Control  (n = 598) | | |  |  |
| **T1** | | | | | | | | | |  |  |
| *Never* | 311 (48.3%) | | 360 (56.3%) |  |  | 252 (37.8%) | 237 (39.6%) | | |  |  |
| *A little* | 163 (25.3%) | | 154 (24.1%) |  |  | 217 (32.6%) | 198 (33.1%) | | |  |  |
| *Sometimes* | 70 (10.9%) | | 34 (5.3%) |  |  | 62 (9.3%) | 77 (12.9%) | | |  |  |
| *A lot* | 42 (6.5%) | | 40 (6.3%) |  |  | 75 (11.3%) | 39 (6.5%) | | |  |  |
| *Always* | 58 (9.0%) | | 52 (8.1%) |  |  | 60 (9.0%) | 47 (7.9%) | | |  |  |
| **T2** |  | |  | 0.013 | .908 |  |  | | | **13.125** | **<.001** |
| *Never* | 390 (62.2%) | | 271 (47.2%) |  |  | 384 (57.7%) | 271 (47.2%) | | |  |  |
| *A little* | 144 (23.0%) | | 175 (30.5%) |  |  | 170 (25.6%) | 175 (30.5%) | | |  |  |
| *Sometimes* | 29 (4.6%) | | 49 (8.5%) |  |  | 42 (6.3%) | 49 (8.5%) | | |  |  |
| *A lot* | 25 (4.0%) | | 41 (7.1%) |  |  | 25 (3.8%) | 41 (7.1%) | | |  |  |
| *Always* | 39 (6.2%) | | 38 (6.6%) |  |  | 44 (6.6%) | 38 (6.6%) | | |  |  |
| **T3** | |  |  | 1.596 | .206 | | |  |  | 1.366 | .242 |
| *Never* | 394 (63.0%) | | 368 (61.2%) |  |  | 328 (49.8%) | 259 (46.1%) | | |  |  |
| *A little* | 129 (20.6%) | | 126 (21.0%) |  |  | 172 (26.1%) | 161 (28.6%) | | |  |  |
| *Sometimes* | 41 (6.6%) | | 46 (7.7%) |  |  | 75 (11.4%) | 72 (12.8%) | | |  |  |
| *A lot* | 37 (5.9%) | | 36 (6.0%) |  |  | 43 (6.5%) | 30 (5.3%) | | |  |  |
| *Always* | 24 (3.8%) | | 25 (4.2%) |  |  | 41 (6.2%) | 40 (7.1%) | | |  |  |

Note: Chi-square and p-value for between groups effect in an ordinal logistic regression with baseline as a covariate.
