# Supplementary material for: A comic-based body image intervention for adolescents in semi-rural Indian schools: A randomised controlled trial
Source: Int J Clin Health Psychol. 2025 Jan 26;25(1):100546. doi: 10.1016/j.ijchp.2025.100546 (PMC11795790; doi:10.1016/j.ijchp.2025.100546)
Supplement: Supplementary file 8 [file mmc8.docx]

S8. Frequency and percentages for body size satisfaction (figure rating scale) at T1, T2, and T3

|  | Girls (n = 1266) | | | | *x^2^* value | *p* value | | Boys (n = 1221) | | | | *x^2^* value | *p* value |
| --- | --- | --- | --- | --- | --- | --- | --- | --- | --- | --- | --- | --- | --- |
|  | Intervention  (n = 630) | | Control  (n = 636) | |  | |  | Intervention  (n = 645) | | Control  (n = 576) | |  |  |
| **T1** | | | | | | | | | | | |  |  |
| *0 (Very satisfied)* | 292 (46.3%) | | 313 (49.2%) | |  | |  | 258 (40.0%) | | 242 (42.0%) | |  |  |
| *1* | 214 (34.0%) | | 205 (32.2%) | |  | |  | 247 (38.3%) | | 202 (35.1%) | |  |  |
| *2* | 70 (11.1%) | | 78 (12.3%) | |  | |  | 83 (12.9%) | | 88 (15.3%) | |  |  |
| *3* | 29 (4.6%) | | 20 (3.1%) | |  | |  | 37 (5.7%) | | 26 (4.5%) | |  |  |
| *4* | 13 (2.1%) | | 14 (2.2%) | |  | |  | 15 (2.3%) | | 13 (2.3%) | |  |  |
| *5* | 6 (1.0%) | | 5 (0.8%) | |  | |  | 4 (0.6%) | | 2 (0.3%) | |  |  |
| *6* | 1 (0.2%) | | - | |  | |  | 1 (0.2%) | | 1 (0.2%) | |  |  |
| *7* | 3 (0.5%) | | 1 (0.2%) | |  | |  | - | | 2 (0.3%) | |  |  |
| *8 (Very dissatisfied)* | 2 (0.3%) | | - | |  | |  | - | | - | |  |  |
| **T2** |  | |  | | 1.585 | | .208 |  | |  | | 0.004 | .952 |
| *0 (Very satisfied)* | 320 (51.6%) | | 283 (47.0%) | |  | |  | 285 (43.2%) | | 234 (41.1%) | |  |  |
| *1* | 214 (34.5%) | | 236 (39.2%) | |  | |  | 237 (36.0%) | | 229 (40.2%) | |  |  |
| *2* | 41 (6.6%) | | 48 (8.0%) | |  | |  | 71 (10.8%) | | 62 (10.9%) | |  |  |
| *3* | 21 (3.4%) | | 21 (3.5%) | |  | |  | 35 (5.3%) | | 29 (5.1%) | |  |  |
| *4* | 15 (2.4%) | | 8 (1.3%) | |  | |  | 17 (2.6%) | | 11 (1.9%) | |  |  |
| *5* | 7 (1.1%) | | 3 (0.5%) | |  | |  | 10 (1.5%) | | 3 (0.5%) | |  |  |
| *6* | 2 (0.3%) | | 3 (0.5%) | |  | |  | 1 (0.2%) | | - | |  |  |
| *7* | - | | - | |  | |  | 2 (0.3%) | | 2 (0.4%) | |  |  |
| *8 (Very dissatisfied)* | - | | - | |  | |  | 1 (0.2%) | | - | |  |  |
| **T3** | |  | |  | 0.374 | | .541 | |  | |  | 3.582 | .058 |
| *0 (Very satisfied)* | 343 (55.0%) | | 324 (51.9%) | |  | |  | 358 (54.4%) | | 286 (51.1%) | |  |  |
| *1* | 216 (34.6%) | | 208 (34.6%) | |  | |  | 243 (36.0%) | | 202 (36.1%) | |  |  |
| *2* | 49 (7.9%) | | 48 (8.0%) | |  | |  | 42 (6.4%) | | 49 (8.8%) | |  |  |
| *3* | 10 (1.6%) | | 16 (2.7%) | |  | |  | 10 (1.5%) | | 22 (3.9%) | |  |  |
| *4* | 3 (0.5%) | | 2 (0.3%) | |  | |  | 3 (0.5%) | | - | |  |  |
| *5* | 2 (0.3%) | | 3 (0.5%) | |  | |  | 2 (0.3%) | | 1 (0.2%) | |  |  |
| *6* | 1 (0.2%) | | - | |  | |  | - | | - | |  |  |
| *7* | - | | - | |  | |  | - | | - | |  |  |
| *8 (Very dissatisfied)* | - | | - | |  | |  | - | | - | |  |  |

Note: Chi-square and p-value for between groups effect in an ordinal logistic regression with baseline as a covariate.
